# Experimental measurement of tympanic membrane response for finite element model validation of a human middle ear

**DOI:** 10.1186/2193-1801-2-527

**Published:** 2013-10-17

**Authors:** Tae-Soo Ahn, Moo-Jin Baek, Dooho Lee

**Affiliations:** School of Mechanical Engineering, Dongeui University, 176, Eumgwangno, Busanjin-gu, Busan 614-714 South Korea; School of Otolaryngology Head and Neck Surgery, Inje University, 875, Zha-dong, Haeundae-gu, Busan 612-896 South Korea

**Keywords:** Laser doppler vibrometer (LDV), Tympanic membrane, Middle ear, Umbo displacement transfer function (UDTF), Finite element model

## Abstract

The middle ear consists of a tympanic membrane, ligaments, tendons, and three ossicles. An important function of the tympanic membrane is to deliver exterior sound stimulus to the ossicles and inner ear. In this study, the responses of the tympanic membrane in a human ear were measured and compared with those of a finite element model of the middle ear. A laser Doppler vibrometer (LDV) was used to measure the dynamic responses of the tympanic membrane, which had the measurement point on the cone of light of the tympanic membrane. The measured subjects were five Korean male adults and a cadaver. The tympanic membranes were stimulated using pure-tone sine waves at 18 center frequencies of one-third octave band over a frequency range of 200 Hz ~10 kHz with 60 and 80 dB sound pressure levels. The measured responses were converted into the umbo displacement transfer function (UDTF) with a linearity assumption. The measured UDTFs were compared with the calculated UDTFs using a finite element model for the Korean human middle ear. The finite element model of the middle ear consists of three ossicles, a tympanic membrane, ligaments, and tendons. In the finite element model, the umbo displacements were calculated under a unit sound pressure on the tympanic membrane. The UDTF of the finite element model exhibited good agreement with that of the experimental one in low frequency range, whereas in higher frequency band, the two response functions deviated from each other, which demonstrates that the finite element model should be updated with more accurate material properties and/or a frequency dependent material model.

## Introduction

The auditory system is a very complex sensory system. It can be categorized into three main parts: the outer ear, middle ear, and inner ear. Each part of the ear has characteristic roles in recognizing sound stimulus in the human brain. The middle ear consists of a tympanic membrane and three ossicles (malleus, incus, and stapes), which are fixed to the temporal bone by ligaments and tendons. The middle ear transfers sound pressure fluctuations in the air into vibrational motions of the cochlear fluid in the inner ear through the impedance matching function of the middle ear between the air and cochlear fluid.

Many studies to develop a numerical dynamic model for the middle ear have been undertaken for pathological or academic purposes (Volandri et al. 2011; Volandri et al. 2012). For a dynamic model for the middle ear, Funnell *et al.* (Funnell and Laszlo 1978) was the first researcher to introduce the finite element (FE) method for a cat’s middle ear. Then, Williams *et al.* (Williams and Lesser 1990) developed a finite element model for a human tympanic membrane. Wada *et al.* (1992) investigated the dynamic behavior of the human middle ear using an FE model that includes the eardrum and ossicles. They considered the cochlear impedance and stiffness of the annular ligament of the tympanic membrane in the FE model. Later, an updated and refined FE model was reported (Koike et al. 2002). Researchers (Sun et al. 2002; Gan et al. 2002; Gan et al. 2006; Zhao et al. 2009) explored the sound transfer characteristics of the middle ear using finite element models and compared these with previous studies.

Another approach identifying the sound transfer characteristics of the middle ear is to directly measure the vibration of the middle ear. A typical apparatus used to measure the middle ear vibration is a laser Doppler vibrometer (LDV). The LDV is a very sensitive non-contact optical measurement system. Goode *et al*. (1993;1996) measured the tympanic membrane displacements in a total of 95 live human ears. Also, the transfer function of the human middle ear was measured for cadaver temporal bones (Gan et al. 2004) and the stapes velocity was directly measured for a cadaver (Voss et al. 2000). Rosowski *et al*. (2003) compared the measured responses of hearing loss subjects with those of normal subjects.

Despite much research being undertaken on the middle ear, it remains a large uncertainty in the development of a dynamic model for the transfer characteristics of the human middle ear. First, the dynamic response of the middle ear varies according to its geometrical structures of the middle ear. This variability primarily arises from the differences of the geometrical structure between individuals (Voss et al. 2008). A recent research (Voss et al. 2008; Shahnaz and Bork 2006) reported significant differences in energy reflectance of middle ear between Caucasians and Chinese. Second, the material properties of the middle ear components, such as the tympanic membrane, joints, tendons, and ligaments, have much uncertainty so that for many components, only wide ranges of the real values can be estimated (Volandri et al. 2011; Volandri et al. 2012). Therefore, in order to develop a valid numerical model of the human middle ear for individuals, the specific geometrical information of the middle ear should be included in the model and the numerical model should be validated using the experimental data. Recently, an FE model of a middle ear based on the anthropometric measure of a Korean male cadaver was developed by the authors and the numerical results were compared with the published experimental data (Gal et al. 2011). However, the experimental data was not for Korean male subjects; therefore, a more refined experimental validation of the FE model of the human middle ear is required for Korean subjects.

In this study, a finite element model for the human middle ear is developed and updated for Korean male subjects. In order to validate the FE model, the dynamic responses of the tympanic membrane were measured using an LDV for Korean male subjects and a Korean male cadaver. Then, the measured dynamic responses of the tympanic membrane were compared with those of the FE model.

## Methods

### Finite element model of the middle ear

**Figure 1:**
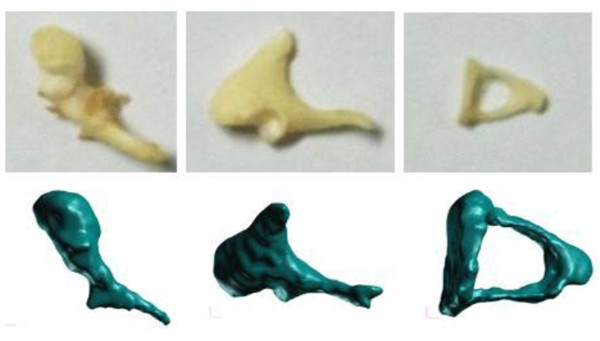
**3D solid models of the ossicles (lower row) compared with the real ossicles (upper row).**

**Figure 2:**
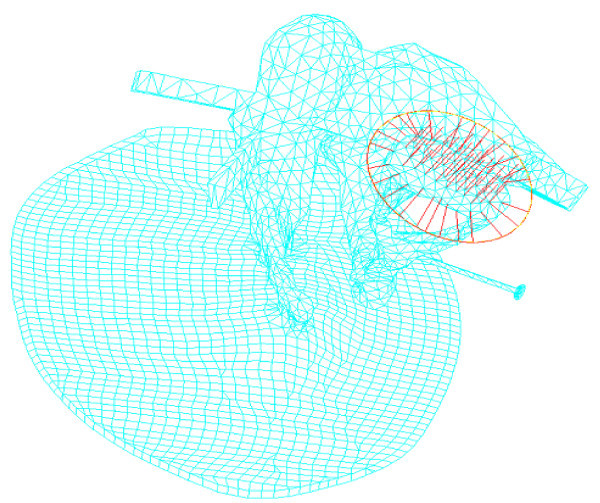
**FE model for the middle ear.**

Developing an FE model for the middle ear is a very challenging issue for two reasons: the geometric modeling and the material properties. First, the geometry of middle ear is a complex three-dimensional solid as well as having very small dimensions. For example, the stapes is the smallest bone in the human body: its length is less than 3 mm and its weight is approximately 3 mg, which makes it very difficult to build the precise geometry of the whole middle ear. Second, the accurate mechanical properties of the biomaterial that comprise the middle ear are not well known, so the FE model has numerous uncertainties in its dynamic responses. Recently, the authors developed an FE model of the middle ear for the transfer function of the middle ear (Gal et al. 2011). For the FE model, the geometry of the ossicles was obtained through micro-CT scanning of the temporal bone of a Korean cadaver, as shown in Figure 1. Figure 2 presents the FE model of the middle ear, which is composed of shell and solid elements. In the FE model, the tympanic membrane and tympanic annular ligament were modeled with shell elements, whereas the ossicles, ligaments, tendons, and muscles of the middle ear were represented as three-dimensional solid elements. The stapes footplate from which the vibrations are transmitted into the motion of the cochlear fluid through an oval window was modeled as 42 elastic springs and viscous dampers for the vertical direction to the footplate. The lateral motion of the footplate was also constrained using linear springs.

In this study, the FE model of Ref. (Gal et al. 2011) was updated in the material properties and was used to calculate the dynamic characteristics of the middle ear. In the material properties of the FE model, two types of material properties were updated: the structural damping of the middle ear and the stiffnesses of the ligaments and joints. First, the structural damping coefficients for the middle ear were added to the FE model, whereas in the previous model the peaky responses were controlled using the proportional damping associated with the system matrices. Another update was the material properties of the incudostapedial joint and anterior malleolar ligament to those that were recently measured from human cadavers (Zhang and Gan 2011; Cheng and Gan 2008). Zhang and Gan (2011) measured the mechanical properties of incudostapedial joints for eight samples harvested from human cadaver temporal bones using a micro MTS, and reported that the incudostapedial joint is a viscoelastic structure with nonlinear stress–strain relationship. They showed that the elastic modulus changed from 0.30 to 8.92 MPa when the stretch ratio increased from 1 to 1.72. However, one can see that the nonlinearity is not so severe in the low stretch region up to 1.2 where the elastic modulus changed 0.3 to 0.7 MPa. In this study, for the elastic modulus of the incudostapedial joint the value at 1.1 stretch ratio was selected, which was 0.43 MPa. Similarly, Cheng and Gan (2008) measured the mechanical properties of anterior malleolar ligament. It was shown in Ref. (Zhang and Gan 2011; Cheng and Gan 2008) that the elastic modulus of anterior malleolar ligament changed 0.22 to 1.2 MPa when the stretch ratio increased from 1 to 1.2. In the modified FE model for the middle ear, we selected 0.46 MPa as the stiffness value of the anterior malleolar ligament which was the value of the measured constitutive equation at stretch ratio 1.1. In Table 1, the material properties used in the FE model are summarized and are compared with the values from the previous studies. Using the modified FE model, the frequency responses of the middle ear were calculated.

**Table 1:** **Material properties used in the middle ear FE model**

Type of properties	Composition	Present study	(Koike et al.^2002^)	Gan et al. (2002, 2006)
Young's modulus (N/m^2^)	Tympanic membrane(tensa)	3.34 × 10^7^	3.34 × 10^7^	3.2 × 10^7^
	Tympanic membrane(flaccida)	1.11 × 10^7^	1.11 × 10^7^	1.0 × 10^7^
	Malleus	1.2 × 10^10^	1.2 × 10^10^	1.41 × 10^10^
	Incus	1.2 × 10^10^	1.2 × 10^10^	1.41 × 10^10^
	Stapes	1.2 × 10^10^	1.2 × 10^10^	1.41 × 10^10^
	Incudomalleolar joint	1.2 × 10^10^	-	1.41 × 10^10^
	Incudostapedial joint	6.0 × 10^5^	6.0 × 10^6^	6.0 × 10^5^
	Anterior malleal ligament	2.1 × 10^7^	2.1 × 10^7^	2.1 × 10^6^
	Posterior incudal ligament	6.5 × 10^5^	6.5 × 10^5^	6.5 × 10^5^
	Tensor tympani muscle	2.6 × 10^5^	2.6 × 10^6^	2.6 × 10^6^
	Manubrium	4.7 × 10^9^	-	4.7 × 10^9^
	Stapedius muscle	5.2 × 10^5^	5.2 × 10^5^	-
	Tympanic annular ligament	6.0 × 10^5^	-	6.0 × 10^5^
Density (kg/m^2^)	Tympanic membrane(tensa)	1.2 × 10^3^	1.2 × 10^3^	1.2 × 10^3^
	Tympanic membrane(flaccida)	1.2 × 10^3^	1.2 × 10^3^	1.2 × 10^3^
	Malleus(head)	2.55 × 10^3^	2.5-6.2 × 10^3^	2.55 × 10^3^
	Malleus(neck)	4.53 × 10^3^	2.5-6.2 × 10^3^	4.53 × 10^3^
	Malleus(handle)	3.70 × 10^3^	2.5-6.2 × 10^3^	3.70 × 10^3^
	Incus(body)	2.36 × 10^3^	2.5-6.2 × 10^3^	2.36 × 10^3^
	Incus(short process)	2.26 × 10^3^	2.5-6.2 × 10^3^	2.26 × 10^3^
	Incus(long process)	5.08 × 10^3^	2.5-6.2 × 10^3^	5.08 × 10^3^
	Stapes	2.2 × 10^3^	2.5-6.2 × 10^3^	2.2 × 10^3^
	Incudomalleolar joint	3.2 × 10^3^	2.5 × 10^3^	3.2 × 10^3^
	Incudostapedial joint	1.2 × 10^3^	2.5 × 10^3^	1.2 × 10^3^
	Anterior malleal ligament	2.5 × 10^3^	2.5 × 10^3^	-
	Posterior incudal ligament	2.5 × 10^3^	2.5 × 10^3^	-
	Tensor tympani muscle	2.5 × 10^3^	2.5 × 10^3^	-
	Manubrium	1.0 × 10^3^	2.5 × 10^3^	1.0 × 10^3^
	Stapedius muscle	2.5 × 10^3^	2.5 × 10^3^	-
	Tympanic annular ligament	2.5 × 10^3^	2.5 × 10^3^	-
Spring constant (N/m)	Stapedius annular ligament	9	-	9
	Cochlear fluid	70(42)	-	60(49)
Poisson’s ratio		0.3	0.3	0.3
Damping parameters (N s/m)	Cochlear fluid	0.054	-	0.054

**Figure 3:**
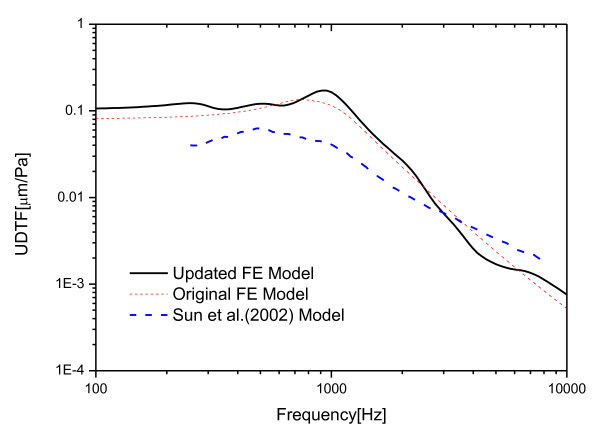
**UDTF compared with the previous FE models.**

For the frequency response function, a uniform unit sound pressure was applied to the outer side of the tympanic membrane. Then, the frequency responses of the displacements on the umbo region were averaged in order to calculate the umbo displacement transfer function (UDTF). Figure 3 presents the UDTF calculated using the modified FE model compared with that of the previous model(Gal et al. 2011) and a published FE model(Sun et al. 2002). The effect of the updated model parameters resulted in a little level change on lower frequency range and slope variation on higher frequency region.

### Experimental verification

The developed FE model has numerous parameters that inherently have many uncertainties, such as the material properties and boundary conditions. In order to verify the middle ear FE model, the UDTFs were measured for *in-vivo* human subjects and an *in-vitro* cadaver, and then they were compared with those obtained using the FE model. For statistical analyses of measurement data, it was assumed that the UDTFs have a log-normal distribution. The mean and standard deviation of the logarithms of the magnitude data were calculated.

The research was followed the Code of Research Ethics of Dongeui University and approved by the Local Ethical Committee (Department of Mechanical Engineering, Dongeui University) and the National Research Foundation of Korea.

### Experimental setup

**Figure 4:**
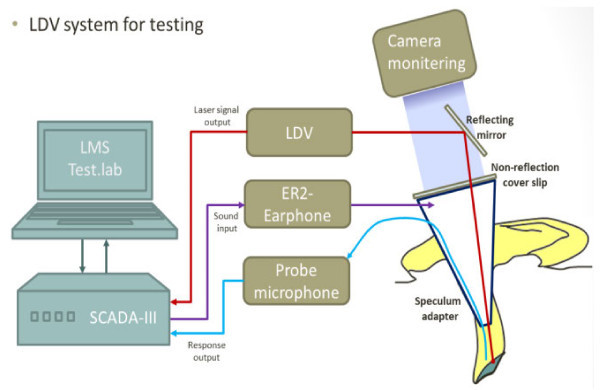
**Schematic diagram of experimental set-up.**

In order to measure the vibrational responses of the tympanic membrane for human subjects, a well-designed apparatus in both response measuring and excitation is required. In this study, the vibrational responses of the tympanic membrane for the *in-vivo* human subjects and an *in-vitro* cadaver were measured using a dedicated experimental setup. Figure 4 shows the schematic diagram of the experimental setup. In the experimental setup, an LDV was used to measure the vibration, an earphone was used to excite the cavity, and a probe microphone was used to measure the sound pressure level in the cavity. All signals were processed in a PC-based system.

**Figure 5:**
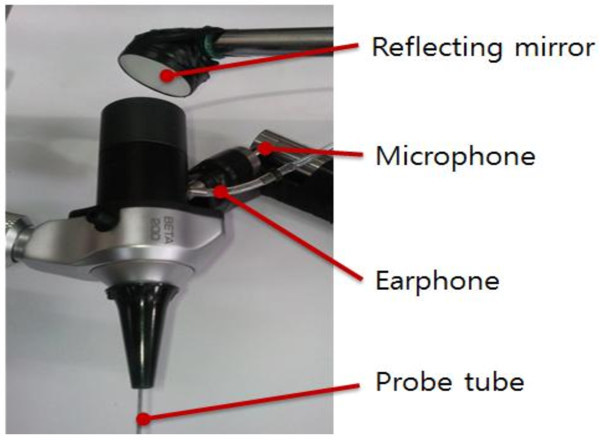
**Speculum module used in the experiment.**

**Figure 6:**
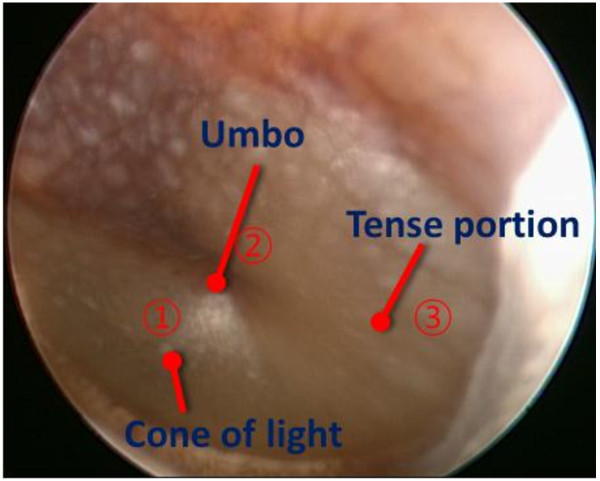
**Measurement points on tympanic membrane.**

**Figure 7:**
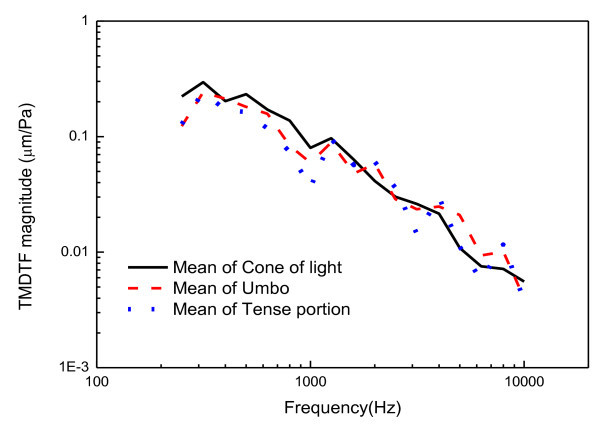
**Measured displacements of the tympanic membrane for a human temporal bone.**

First, the LDV is the most popular measurement system for middle ear because the tympanic membrane is so thin that any contact sensors cause significant perturbation in the dynamic behavior of the middle ear. For the LDV measurement, an EM4SYS LSV-110D laser vibrometer was used: it had a helium-neon laser beam with a laser spot size of less than 200 μm, 20 ~ 40,000 Hz frequency range, and a maximum 1 mW laser beam output power. In order to sustain the laser path in the ear canal, a speculum device was used in the experiment as shown in Figure 5. The helium-neon laser beam was guided into the speculum module using a mirror and it was directed to a point on the tympanic membrane. An ER-2 earphone and a GRAS probe microphone were used to excite the cavity and measure the sound field, respectively. The earphone generated a pure tone sound signal in order to excite the ear canal cavity at a prescribed level. The sound pressure level in the ear cavity controlled with 60 or 80 dB in magnitude. The probe microphone tube, which has a diameter of 1 mm, was installed so that the passage of the laser beam was not obstructed. The earphone and microphone were fixed on the speculum module, which was sealed by a non-reflective transparent glass in order to maintain the sound pressure level in the ear canal space constant during the excitation. The speculum module was inserted in the ear canal as illustrated in Figure 4. During the excitation, the velocity responses of the tympanic membrane were measured using a SCADAS-III front-end and the data acquisition software, LMS Test.Lab (LMS). In order to verify the response variation across the points on the tympanic membrane, three points (umbo, tense portion, and cone of light) were selected and the dynamic responses at these points were measured for a human temporal bone. Figure 6 shows the selected locations on the tympanic membrane; Figure 7 shows the response variation between the three locations on the tympanic membrane. Naturally, the responses of the tympanic membrane vary smoothly from point to point due to its flexible mode. However, in Figure 7, it can be seen that the variation of the frequency responses among the points was not significant and the responses at the umbo and the cone of light exhibited similar dynamic characteristics. It is noteworthy that the cone of light region exhibited the largest reflectivity for the laser beam on the tympanic membrane, which leads to a high quality signal conditioning. Thus, the cone of light was selected as the measurement location instead of the umbo point. For each subject, the sound pressure responses were measured 20 times and averaged at each frequency.

### *In-vivo* measurement

**Figure 8:**
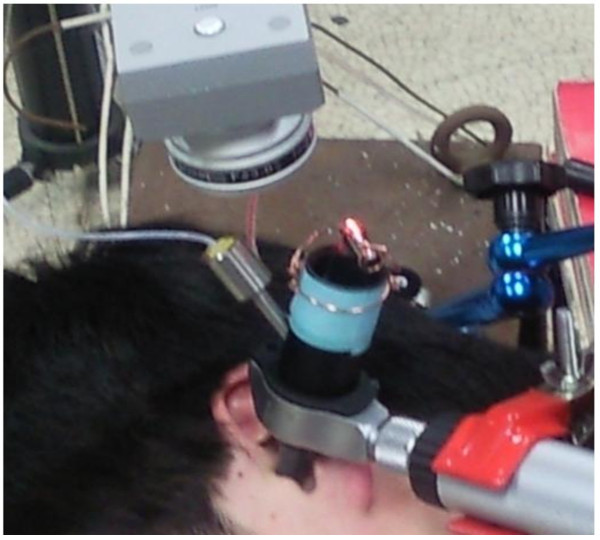
**Experimental set-up for in-vivo measurement installed on a human subject.**

In order to perform the experimental measurement for the *in-vivo* tympanic membrane, human subjects were recruited and examined for their physical condition. Five male subjects with ages between 25 and 35 years were recruited. The subjects had neither hearing loss nor scars on their tympanic membranes, which were verified with an otoscope check. In the *in-vivo* measurement, the movements of the live subject during the data acquisition cause severe noisy signal problems. Therefore, the movement of subject should be minimized during the experiment. In the *in-vivo* experiments, the subjects laid on their side as comfortable as possible on a test bed and their head rested in a headrest as shown in Figure 8. However, it should be noted that a little movement is inevitable in the in vivo measurement, e.g. due to the heartbeat, so a noisy signal cannot be avoided. The speculum module was installed in the ear of each subject. The lengths of the probe microphone and earphone were controlled in order to maintain a 5 mm gap between the end of the microphone and the tympanic membrane. The magnitude of the excitation was adjusted in order to maintain a constant sound pressure level in the ear cavity at each frequency. The subjects were requested to hold their breath during the data acquisition. The measurements were repeated frequency-by-frequency at the center frequencies of one-third octave bands from 200 to 10,000 Hz. For each human subject, the measurements were repeated for two excitation levels of 60 and 80 dB.

### *In-vitro* measurement

**Figure 9:**
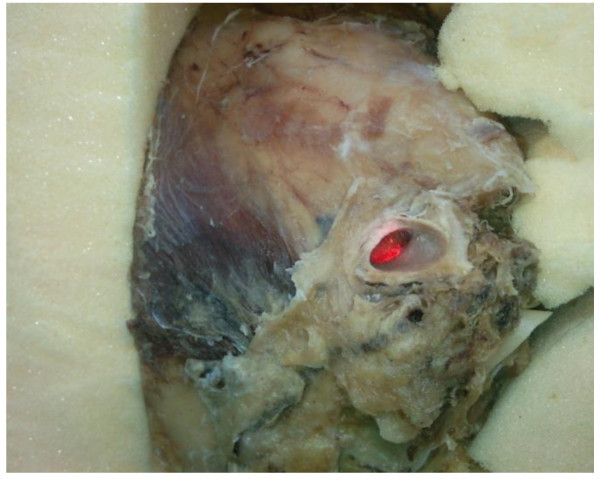
**A temporal bone for in-vitro experiment.**

For comparison, the measurement of the dynamic responses of tympanic membrane was repeated for a human temporal bone that was removed from a Korean male cadaver. The temporal bone had the original skin, tympanic membrane, muscles, tendons, ligaments, and ossicles. The temporal bone was verified using three-dimensional X-ray scanning in order to confirm that the temporal bone did not have a pathological disorder. In order to maintain the fresh condition of the temporal bone, the bone was immersed in saline solution with 2% formalin and refrigerated at 5°C until use. For the measurement, the temporal bone was fixed to a base that was made of a sound absorbing material as shown in Figure 9. The speculum that was used in the *in-vitro* experiment was installed in the ear canal of the temporal bone and the dynamic responses of the tympanic membrane were obtained using the same test conditions as those of the *in-vitro* experiment.

## Results and discussions

**Figure 10:**
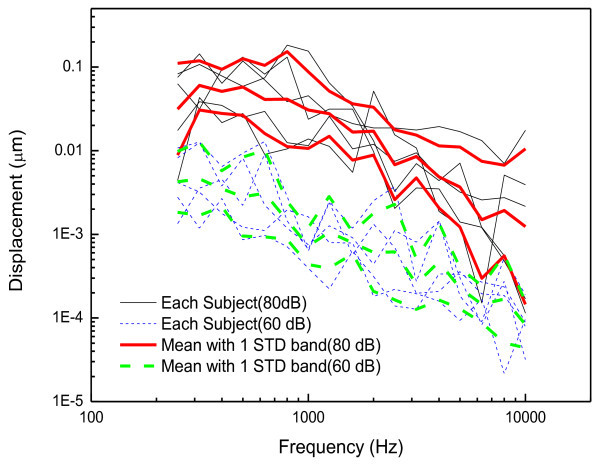
**Displacements of tympanic membrane measured in the in-vivo experiment.**

**Figure 11:**
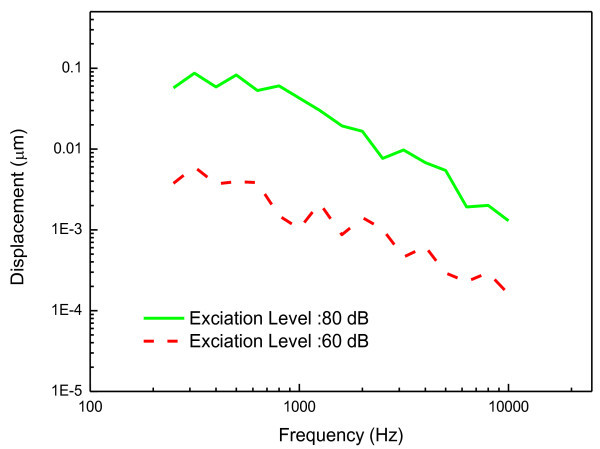
**Displacements of tympanic membrane measured in the in-vitro experiment.**

**Figure 12:**
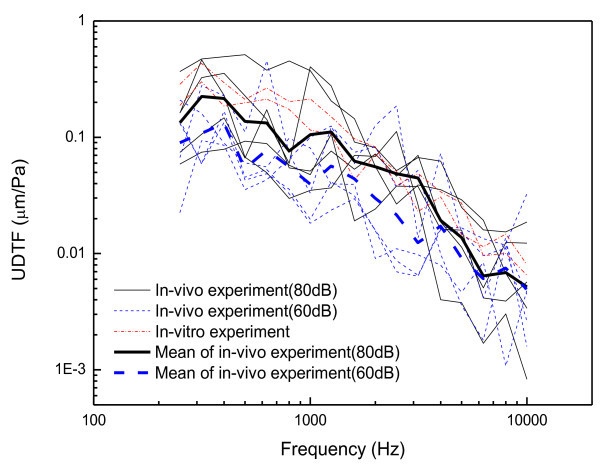
**UDTFs of tympanic membrane calculated from the in-vivo and in-vitro experiments.**

The tympanic membrane displacements measured in the *in-vivo* and *in-vitro* experiments according to the prescribed sound pressure levels are plotted in Figures 10 and 11, respectively. Figure 10*a*lso shows the mean and one standard deviation band according to the excitation levels in the *in-vivo* experiment. The dynamic characteristics of the measured displacements agree well with the general behavior of the tympanic membrane, i.e. the frequency response below 1 kHz is nearly flat and above 1 kHz decreases with the frequency (Volandri et al. 2011). It can be seen that the displacements of the tympanic membrane are proportional to the sound pressure level of the cavity, which indicates that the dynamic behavior of the tympanic membrane can be treated as a linear system for those sound input levels. In order to demonstrate the linearity of the tympanic membrane displacement, the umbo displacement transfer function (UDTF), which is defined as the transfer function due to the unit sound pressure on the tympanic membrane, was calculated for the experimental results. Figure 12 presents the UDTFs converted from the experimental results including the mean UDTFs from each excitation level as well as individual UDTFs. The calculated UDTFs have a very similar pattern to each other in the frequency response, but also have a slight level difference. However, one can see in Figures 10 and 12 that the level differences in the mean UDTFs for each excitation level are smaller than the variation of the UDTFs for each individual. The level differences do not primarily arise from the excitation levels, but rather from the variability between the individual human subjects, which demonstrates that the linearity of the tympanic membrane response over the excitation levels.

**Figure 13:**
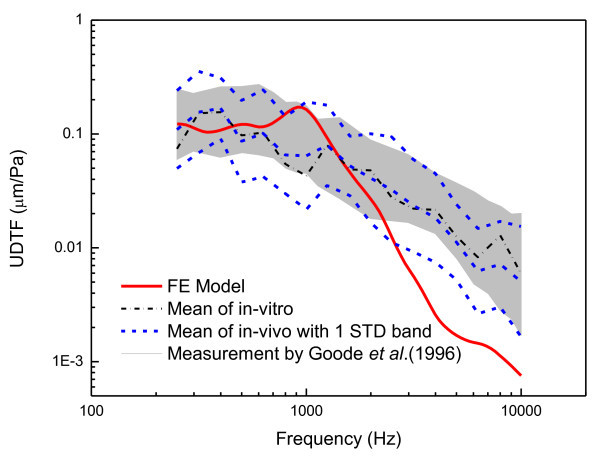
**Calculated UDTF of the FE model compared with measurements.**

The *in-vivo* and *in-vitro* experimental results do not exhibit significant differences, which has been confirmed repeatedly by numerous researchers (Goode et al. 1996; Goode et al. 1993). In this research, the temporal bone used in the experiment was kept for several months before the experiment was performed. Therefore, it is clear that the degradation of the tissue freshness from a dynamic response viewpoint is very slow if the preservation conditions are suitable. Figure 13 summarized the measured UDTFs and the calculated UDTF. The gray region represents the 10th and 90th percentiles of the measured responses from Ref. (Goode et al. 1996) in which 95 human subjects (all Caucasians) were measured. Comparing the UDTFs of the Korean male subjects measured in this study with those of a previous study (Goode et al. 1996), the dynamic behavior of the tympanic membrane for the Korean males appears to fall in the variability range between the 10th and 90th region of the Caucasian in most frequency ranges. However, the presented results cannot eliminate the possibility that the Korean has a little different transfer function characteristics compared with the Caucasian because of the small number of samples and relatively large variations. Recently, a research for Chinese (Shahnaz and Bork 2006) reported significant differences in power reflectance of tympanic membranes in Caucasians and Chinese. In order to verify the difference of the dynamic response for the Korean, further extensive data collection is required.

Finally, in order to verify the validity of the middle ear FE model, the UDTF of the FE model was compared with those of the experimental UDTFs in Figure 13. Here, it should be noted that the individual ear which was modeled by the FE model is not the same with those of experimental subjects. Thus, the comparison should be focused on significant deviations because small difference could be originated from the small structural variation of the middle ear structure. The FE model provides a nearly flat frequency response below the 1 kHz frequency band and exhibits a resonance at near 1 kHz, which is a generally accepted behavior characteristic of the tympanic membrane (Volandri et al. 2011). Figure 13 demonstrates that the FE model describes the dynamic responses below the 2 kHz frequency band well, although the FE model response exhibits a slight level difference in the lower frequency region when compared with those of the Korean subjects. However, it can also be seen that the results of the FE model deviate more and more from the experimental results as the frequency band increases. The reason of the deviation of the FE model at the higher frequency range is not as clear: it can arise from the uncertain material properties and boundary conditions, or from the nonlinearity and/or frequency-dependency of the tissue materials. As a recent measurement for some components of the middle ear reported in Refs. (Zhang and Gan 2011; Cheng and Gan 2008; Cheng et al. 2007), the materials exhibited a large stiffness for a high stress state but a lower stiffness for a lower stress state, which may cause the deviation at the high frequency range because the determinations of operational stress states for the each component of the middle ear are very complex and have large uncertainty. Therefore, the model performance could be enhanced by introducing more precise material properties obtained from direct measurements or indirect identification techniques such as, for example, a statistical calibration method. Another possibility is to develop a frequency-dependent material model for the critical components of the middle ear, such as the joints and ligaments. In summary, the comparison results demonstrate both the usefulness of the FE model and the necessity of updating the numerical model. In the near future, the calibration and updating of the FE model will be carried out.

## Conclusions

The tympanic membrane responses for Koreans were measured with *in-vivo* and *in-vitro* experimental setups using an LDV and the dedicated speculum module. The measured UDTFs were compared with those of the FE model for a Korean subject. From these results, the followings can be concluded.

1. The tympanic membrane responses measured from the *in-vivo* and *in-vitro* experimental setups for Koreans exhibit similar patterns with each other and are in the variability range of the published data. However, the presented results cannot eliminate the possibility that the Korean has a little different transfer function characteristics compared with Caucasians because of the small number of samples and relatively large variations in the measurements.
2. The displacement of the tympanic membrane can be treated as a linear system for the input sound pressure level for moderate magnitudes. The measured frequency responses also exhibited large variations between subjects.
3. The FE model provides valid responses of the tympanic membrane in below 2 kHz. However, the dynamic responses of the FE model decrease more rapidly than the experimental results for the higher frequency region.
4. The uncertainty of the material properties and boundary conditions for the middle ear FE model must be identified quantitatively in order to enhance the performance of the FE model. Furthermore, the frequency dependency of the material properties should be considered, which will be further investigated in future research.

## References

[CR1] Cheng T, Dai C, Gan R (2007). Viscoelastic Properties of Human Tympanic Membrane. Ann Biomed Eng.

[CR2] Cheng T, Gan R (2008). Mechanical properties of anterior malleolar ligament from experimental measurement and material modeling analysis. Biomech Model Mechanobiol.

[CR3] Funnell WRJ, Laszlo CA (1978). Modeling of the cat eardrum as a thin shell using the finite-element method. J Acoust Soc Am.

[CR4] Gal YM, Baek M-J, Lee D (2011). Finite Element Analysis of Sound Transfer Characteristics for Middle Ear. Transac Korean Soc Mechanic Eng.

[CR5] Gan RZ, Sun Q, Dyer RKJ, Chang K-H, Dormer KJ (2002). Three-dimensional Modeling of Middle Ear Biomechanics and Its Applications. Otol Neurotol.

[CR6] Gan RZ, Sun Q, Feng B, Wood MW (2006). Acoustic-structural coupled finite element analysis for sound transmission in human ear--Pressure distributions. Med Eng Phys.

[CR7] Gan RZ, Wood MW, Dormer KJ (2004). Human Middle Ear Transfer Function Measured by Double Laser Interferometry System. Otol Neurotol.

[CR8] Goode RL, Ball G, Nishihara S, Nakamura KG (1996). Laser Doppler vibrometer (LDV): A new clinical tool for the otologist. Am jotol.

[CR9] Goode RL, Ball G, Nishihara S (1993). Measurement of Umbo Vibration in Human Subjects-Method and Possible Clinical Applications. Otol Neurotol.

[CR10] Koike T, Wada H, Kobayashi T (2002). Modeling of the human middle ear using the finite-element method. J Acoust Soc Am.

[CR11] Rosowski JJ, Mehta RP, Merchant SN (2003). Diagnostic Utility of Laser-Doppler Vibrometry in Conductive Hearing Loss with Normal Tympanic Membrane. Otol Neurotol.

[CR12] Shahnaz N, Bork K (2006). Wideband Reflectance Norms for Caucasian and Chinese Young Adults. Ear Hear.

[CR13] Sun Q, Gan RZ, Chang KH, Dormer KJ (2002). Computer-integrated finite element modeling of human middle ear. Biomech Model Mechanobiol.

[CR14] Volandri G, Di Puccio F, Forte P, Carmignani C (2011). Biomechanics of the tympanic membrane. J Biomech.

[CR15] Volandri G, Di Puccio F, Forte P, Manetti S (2012). Model-oriented review and multi-body simulation of the ossicular chain of the human middle ear. Med Eng Physics (0).

[CR16] Voss SE, Horton NJ, Woodbury RR, Sheffield KN (2008). Ear & Hearing.

[CR17] Voss SE, Rosowski JJ, Merchant SN, Peake WT (2000). Acoustic responses of the human middle ear. Hear Res.

[CR18] Wada H, Metoki T, Kobayashi TG (1992). Analysis of dynamic behavior of human middle ear using a finite-element method. J Acoust Soc Am.

[CR19] Williams KR, Lesser THJ (1990). A finite element analysis of the natural frequencies of vibration of the human tympanic membrane. Part I. Br J Audiol.

[CR20] Zhang X, Gan R (2011). Experimental measurement and modeling analysis on mechanical properties of incudostapedial joint. Biomech Model Mechanobiol.

[CR21] Zhao F, Koike T, Wang J, Sienz H, Meredith R (2009). Finite element analysis of the middle ear transfer functions and related pathologies. Med Eng Physics.

